# Circulating Fibroblast Growth Factor 21 (Fgf21) as Diagnostic and Prognostic Biomarker in Renal Cancer

**DOI:** 10.4172/2155-9929.S2-015

**Published:** 2016-05-20

**Authors:** ME Knott, JN Minatta, L Roulet, G Gueglio, L Pasik, SM Ranuncolo, M Nuñez, L Puricelli, MS De Lorenzo

**Affiliations:** 1 Instituto de Oncología “Ángel H Roffo”, Universidad de Buenos Aires (UBA), Argentina; 2 Hospital Italiano de Buenos Aires- Buenos Aires, Argentina; 3 Facultad de Farmacia y Bioquímica UBA, Argentina; 4 Department of Cell Biology and Molecular Medicine, New Jersey Medical School, Rutgers, State University of New Jersey, USA

**Keywords:** Serum fibroblast growth factor 21, Clear cell renal cell carcinoma, Prognosis biomarker

## Abstract

**Background:**

The finding of new biomarkers is needed to have a better sub-classification of primary renal tumors (RCC) as well as more reliable predictors of outcome and therapy response. In this study, we evaluated the role of circulating FGF21, an endocrine factor, as a diagnostic and prognostic biomarker for ccRCC.

**Materials and Methods:**

Serum samples from healthy controls (HC), clear cell and chromophobe RCC cancer patients were obtained from the serum biobank “Biobanco Público de Muestras Séricas Oncológicas” (BPMSO) of the “Instituto de Oncología “Ángel H. Roffo”. Serum FGF21 and leptin were measured by ELISA while other metabolic markers were measured following routinely clinical procedures.

**Results:**

One of our major findings was that FGF21 levels were significantly increased in ccRCC patients compared with HC. Moreover, we showed an association between the increased serum FGF21 levels and the shorter disease free survival in a cohort of 98 ccRCC patients, after adjustment for other predictors of outcome.

**Conclusion:**

Our results suggest that higher FGF21 serum level is an independent prognostic biomarker, associated with worse free-disease survival.

## Introduction

Primary renal tumors (RCC) comprise a heterogeneous group of entities with diverse morphological and molecular characteristics, with variable and unpredictable clinical outcomes [1]. Clinicians face important pitfalls in the treatment of RCC, such as absence of symptoms in early stages of the disease, its high metastatic potential and its resistance to conventional therapy. The finding of new biomarkers is needed for a better sub-classification of RCC tumors as well as reliable predictors of outcome and therapy response.

Clear cell renal cell carcinoma (ccRCC) is the most frequent malignant neoplasm of the renal parenchyma [2,3] and is a cell metabolism disease because metabolizes glucose mostly via glycolysis over Krebs cycle and oxidative phosphorylation [4,5].

Fibroblast growth factor 21 (FGF21) is a hepatic atypical member of the FGF ligand family, which together with the ileal FGF15 (human FGF19) and the bone-produced FGF23 participate in maintaining the whole-body homeostasis. FGF21 is produced in the liver, adipocytes and skeletal muscle and regulates glucose, energy and lipid metabolism [6-10]. FGF21 is a stress-responsive hepatokine which is induced in the liver as a response to injury, regeneration, hepatocarcinogenesis [11] and during kidney dysfunction [12]. FGF21 is primarily metabolized in the kidney and renal secretion is the major route of FGF21 elimination. Its plasma levels increase according to the decline in renal function in patients with chronic and acute renal dysfunction [12].

Moreover, the inter-organ cross-talk secretion from the hepatic FGF21 to adipocytes leads to the correction of deranged glucose, lipid and energy metabolism which benefit the organism during stress-induced pathologies such as obesity, diabetes, fatty liver and infection [13,14].

Administration of FGF21 in diet-induced obese mice exerts health beneficial properties apparently due to an increased production of adiponectin by the adipocytes [15]. Intriguingly, transgenic mice overexpressing FGF21 exhibit a significant increase in mean and maximal lifespan of around 30% due, in part, to the FGF21 inhibitory effect on growth hormone induced IGF-1 hepatic expression [16,17]. Also, genetic ablation of FGFR4 delayed mammary tumor progression by decreasing IGF-1, IGFBP-1 and increasing FGF21 [18]. Elevated serum FGF21 was found in patients with higher Body Mass Index (BMI) values, type 2 diabetes mellitus, metabolic syndrome, coronary heart disease, chronic and acute renal dysfunction, hepatitis, liver fatty degeneration and cirrhosis [12,19,20].

Even though FGF19, FGF21 and FGF23 are all endocrine factors [19], based on the effects of FGF21 on fuel energy, oxidative stress, life-span and its pleiotropic metabolic actions; investigators have suggested that FGF21 is a very promising molecule with therapeutic potential to combat cancer [7,19]. However, up to date, little is known about the role of FGF21 in cancer initiation and progression. Even less is known on the role of circulating levels of FGF21, FGF19 or FGF23 as a cancer biomarker. Based on this we decided to evaluate the role of serum FGF21 levels as a biomarker for ccRCC. Our results indicated that serum FGF21 might be potentially useful as a diagnostic biomarker for ccRCC in combination with other clinical or molecular parameters. Moreover, in our cohort of ccRCC patients, high levels of FGF21 showed to be an independent prognostic biomarker, associated with worse disease-free survival.

## Materials and Methods

### Population

Blood samples were drawn from healthy controls (HC) and eligible renal cancer patients. Subjects were 18 years or older, with a Karnofsky performance score [21] of 80 or higher and with adequate bone marrow, kidney, and liver function. We excluded patients with any of the following criteria: any current treatment, medical history or uncontrolled disorder other than the malignant disease (e.g. alcoholism, medical or psychiatric condition). Table 1 summarizes some features of the studied populations.

Clear cell RCC was graded according to the Fuhrman system [22]. TNM staging was determined using a collaborative stage approach, revised according to the 7th edition of the American Joint Committee on Cancer Staging Manual. We have included 37 Stage (S) I, 14 SII, 20 SIII and 25 SIV ccRCC patients. In addition, we included 10 (SI), 3 (SII) and 1 (SIV) RCC chromophobe patients. Data on pathological findings and clinical follow-up (Md (range): 24 (3-54) months) were obtained from medical charts. All patients who died had clear evidence of uncontrolled tumor growth at the time of death.

The institutional review boards (Instituto de Oncología “Ángel H Rofo” (IOAHR) and “Hospital Italiano de Buenos Aires”) approved the protocol. The studies were done in accordance with the ethical principles outlined in the Declaration of Helsinki and in compliance with the international conference on harmonization good clinical practice guidelines. Every patient provided written informed consent before study-related procedures were done.

### Serum samples

Serum samples were obtained from the serum biobank “Biobanco Público de Muestras Séricas Oncológicas” (BPMSO) of the IOAHR. According to the BPMSO standard procedure, 20 ml of blood were collected in tubes without any anticoagulant and left 15 min at 25°C to allow the clot formation and centrifuged at 600 × g for 10 min. Then, serum was aliquoted and stored at -80°C. Sera aliquots were used only once after thawing. Blood samples were drawn before surgery from untreated patients.

### Serum protein dosages

Serum FGF21 levels were determined using R&D System^®^ colorimetric ELISA test (Minneapolis, MN. Catalog Number: DF2100), following manufacturer's instructions. The samples variation intra- and inter-assay coefficients were 13% and 10% respectively. The minimum detectable dose (MDD) of FGF21 ranged from 1.61 to 8.69 pg/ml with a mean of 4.67 pg/ml. The ELISA was specific for human FGF21 with no cross-reactivity with human FGF19 and FGF23.

Serum Leptin levels were determined using R&D System^®^ ELISA test (Catalog Number: DLP00) following manufacturer's instructions. The samples variation intra- and inter-assay coefficients were 3% and 8% respectively. The MDD of Leptin was lower than 7.8 pg/ml. FGF21 and Leptin concentrations were calculated from the standard calibration curve provided with the kits.

After fasting for 12 hours, the subjects underwent the following laboratory blood analysis: glucose, total cholesterol, high-density lipoprotein cholesterol (HDL), low-density lipoprotein cholesterol (LDL) and triglycerides (TG). We have analyzed the renal function by calculating the clearance of creatinine using Cockcroft-Gault formula. The central laboratory at the IOAHR measured those levels in sera from donors following standards measures and using the Cobas C311 equipment (Roche ^®^).

## Statistical Analysis

List wise method was used to manage missing data. Shapiro-Wilks and Kolmogorov-Smirnov tests were used to evaluate normal distribution of data. Differences in the level of FGF21 among the groups were compared using the Kruskall-Wallis (KW) and Mann-Whitney (MW) tests, appropriate median tests for even skewed data. Statistical correlations between the variables under study were assessed with Spearman's rank correlation coefficient [23].

A receiver operator characteristic (ROC) curve [24] was developed to determine the optimal reference value to differentiate patients from controls and employed to determine sensitivity and specificity. This reference value discriminated between “negative” and “positive” serum FGF21 values.

To analyze the associations between FGF21 and clinico-pathological variables, FGF21 values of ccRCC patients were dichotomized into “low” and “high” expression groups, employing a second cut-off value. The Chi square-test was used to assess statistical significance in bivariate comparisons.

Disease-free survival (DFS) and overall survival (OS) were measured from the date of surgery of primary tumor to the manifestation of local recurrence or death, respectively, or the last follow-up visit. The Kaplan-Meier method was used to analyze DFS and OS. Multivariate analysis was performed, using the stepwise Cox proportional hazards model, to evaluate the prognosis power of each variable independently of the others. Classification tree was performed as another multivariate analysis to find any association among clinico-pathological parameters. In this method, the nodes represent variables which, sequentially, divide data according to a cut off value.

All statistical tests were 2-sided and p<0.05 was considered statistical significant. Data were analyzed using SPSS^®^ version 18 for Windows software package and InfoStat version 2015.

## Results

### Serum FGF21 in healthy controls

**Figure 1:**
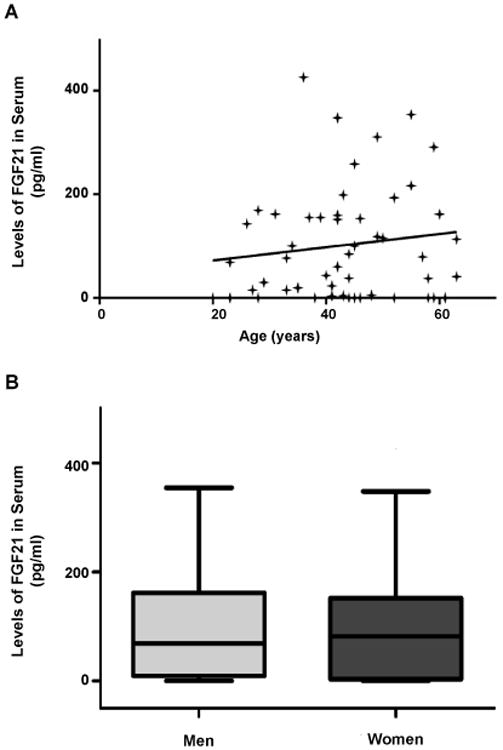
Association of the serum FGF21 levels with age (A) and gender (B) in the healthy control (HC) population.

We evaluated the circulating levels of FGF21 in human HC (n=51) and its association with age and gender to rule out undesired variations. No association was observed between FGF21 serum concentration and gender of HC (MW test) or age (Spearman's rank correlation test) (Figure 1). In addition, HC population was dichotomized into those who had 60 years or more (≥ 60) and those who had less than 60 years (<60) at the moment of the blood drawn. No statistical difference was observed between these groups (data not shown).

**Figure 2:**
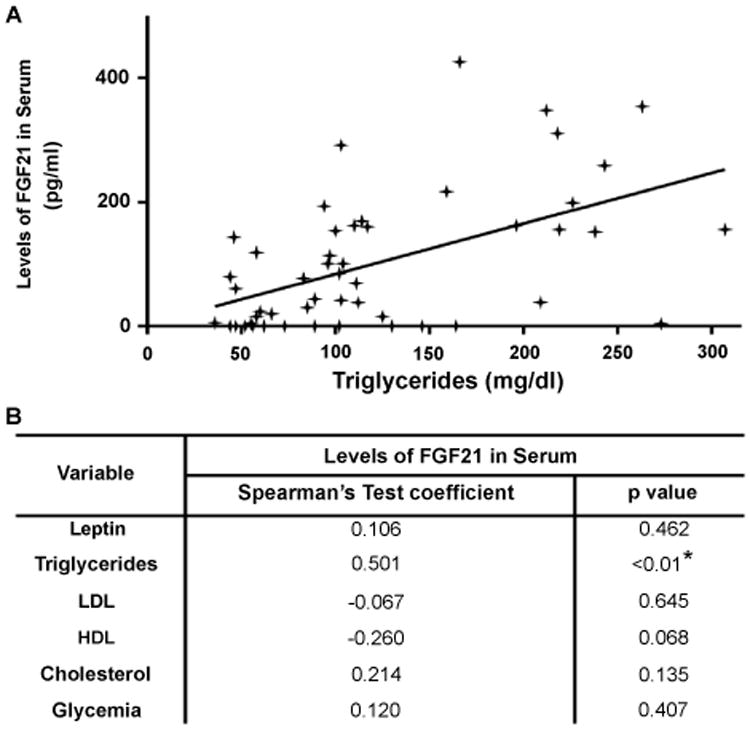
Spearman's rank correlation between the serum FGF21 levels and metabolic parameters in the healthy control (HC) population.

We also analyzed whether there was a potential association among the circulating levels of serum FGF21, lipid profile or leptin concentration which regulates the body fat storage. Interestingly, FGF21 levels significantly correlated with the levels of triglycerides (Figure 2) (Spearman's rank correlation test r=0.50, p <0.01), but there was no correlation between FGF21 and LDL, HDL, cholesterol or glycemia (Figure 2). In a multiple stepwise linear regression analysis, where the FGF21 serum levels were the dependent variable, triglycerides remained independently associated with FGF21 after the adjustments performed for age, sex and other biochemical parameters (Beta coefficient: 0.60, p=0.005).

### Serum FGF21 in ccRCC patients

**Figure 3:**
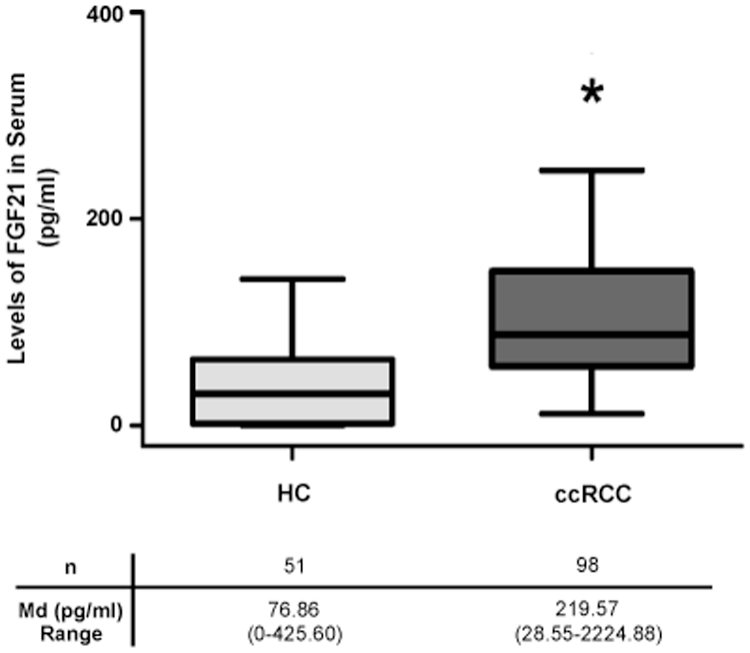
Serum FGF21 levels in healthy controls (HC) and patient populations (ccRCC). (Kruskal Wallis test: p<0.0001). Md, median.

**Figure 4:**
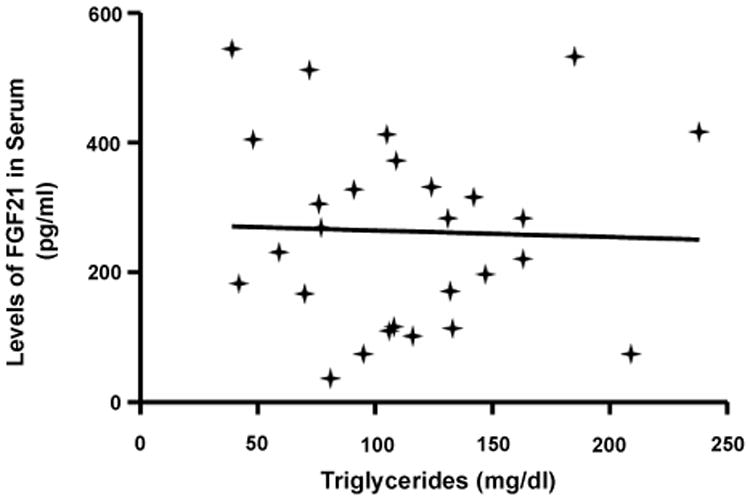
Serum FGF21 levels showed no association with the triglycerides levels. (Spearman's rank correlation test, NS).

Interestingly, patients with ccRCC had significantly higher levels of serum FGF21 compared with HC (n=98, KW test: p<0.0001) (Figure 3). In our cohort of patients, serum FGF21 levels were not associated with the body weight, BMI, lipid profile, glycemia, leptin or the creatinine clearance. In addition, serum FGF21 levels showed no association with the triglycerides levels (Spearman's rank correlation test, NS) (Figure 4).

Using the ROC curve analysis, we determined that 130.64 pg/ml of serum FGF21 was the reference value, close to the inflection point on the curve (data not shown), which maximized sensitivity and specificity. The positive and negative serum FGF21 values were defined based on this reference value. In our population the sensitivity to diagnose ccRCC was 80.61%, while the specificity was 64.61%. In other words, 79/98 ccRCC patients had positive values, whereas only 18/51 of HC had.

### Serum FGF21 and ccRCC's clinico-pathological parameters

**Figure 5:**
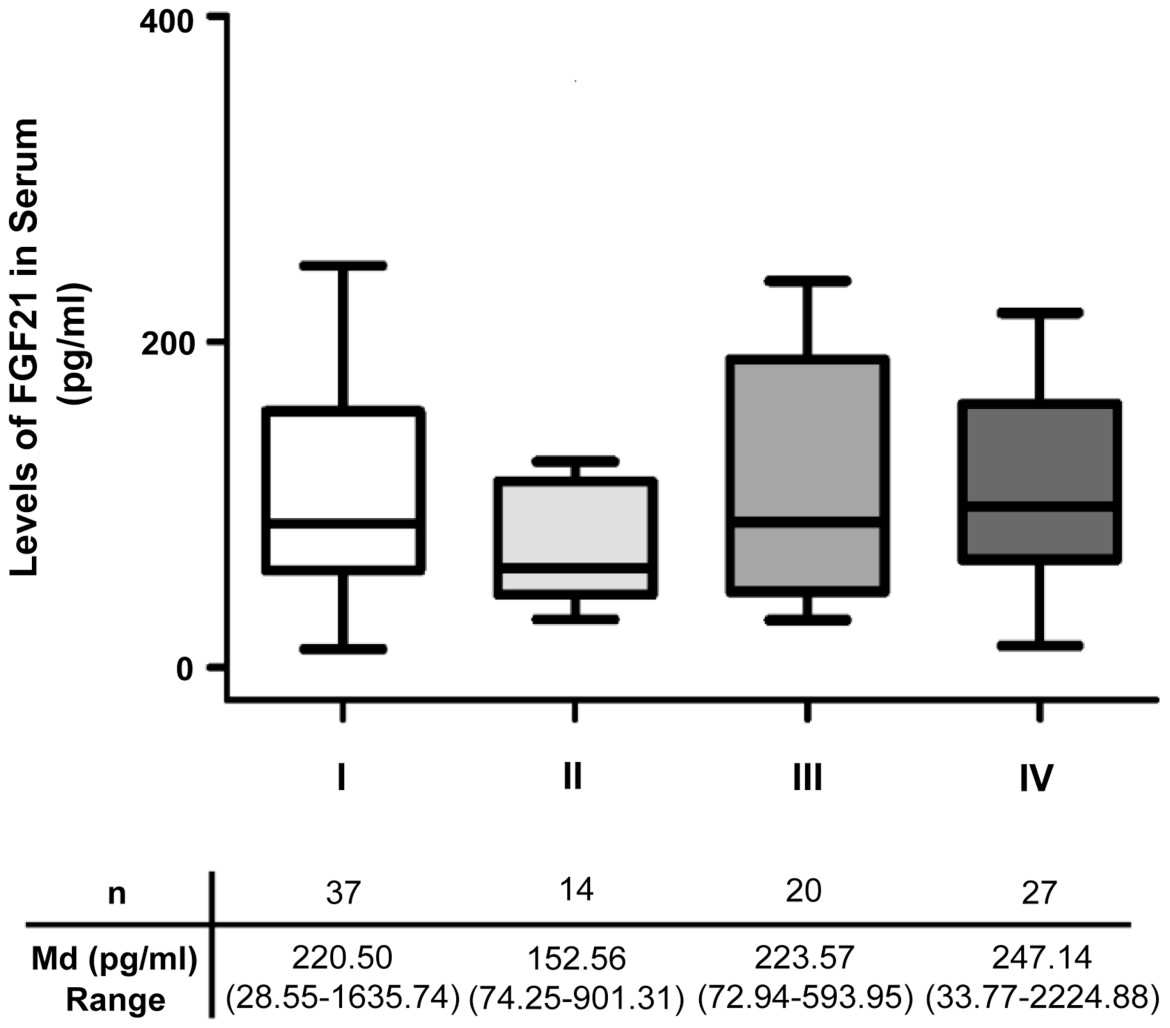
Serum FGF21 levels among the different stages (I to IV) of the ccRCC patients. (Kruskal Wallis test, p=0.44).

No differences in serum FGF21 levels were observed among the various ccRCC stages (KW test, p=0.44) (Figure 5). Moreover, the amount of circulating FGF21 was significantly increased since the earliest stages of the disease. To gain further insights into the clinical relevance of circulating FGF21 in ccRCC patients, we analyzed possible associations between FGF21 and some clinico-pathological parameters (Table 2). For this purpose FGF21 values were dichotomized into “low” or “high” using 219.57 pg/ml (50th percentile of serum FGF21 concentration from ccRCC patients) as cut-off point. No significant association was observed with age, sex, obesity, known risk factors (smoking, obesity or hypertension), performance status, Stage, Fuhrman's nuclear grade, tumor size or distant metastases (*Chi square* test, NS). In addition, serum FGF21 levels showed no association with the triglycerides levels, dichotomized into “high” or “normal” applying the cut-off value of 200 mg/dl (*Chi Square* test, NS) (Table 2).

### Serum FGF21 and ccRCC patient's survival

In our cohort of ccRCC patients the OS rate was 100% for Stage I and 41.6% for Stage IV, and this classification predicted survival reliably. No significant association was found between low and high serum FGF21 categories and OS (data not shown).

**Figure 6:**
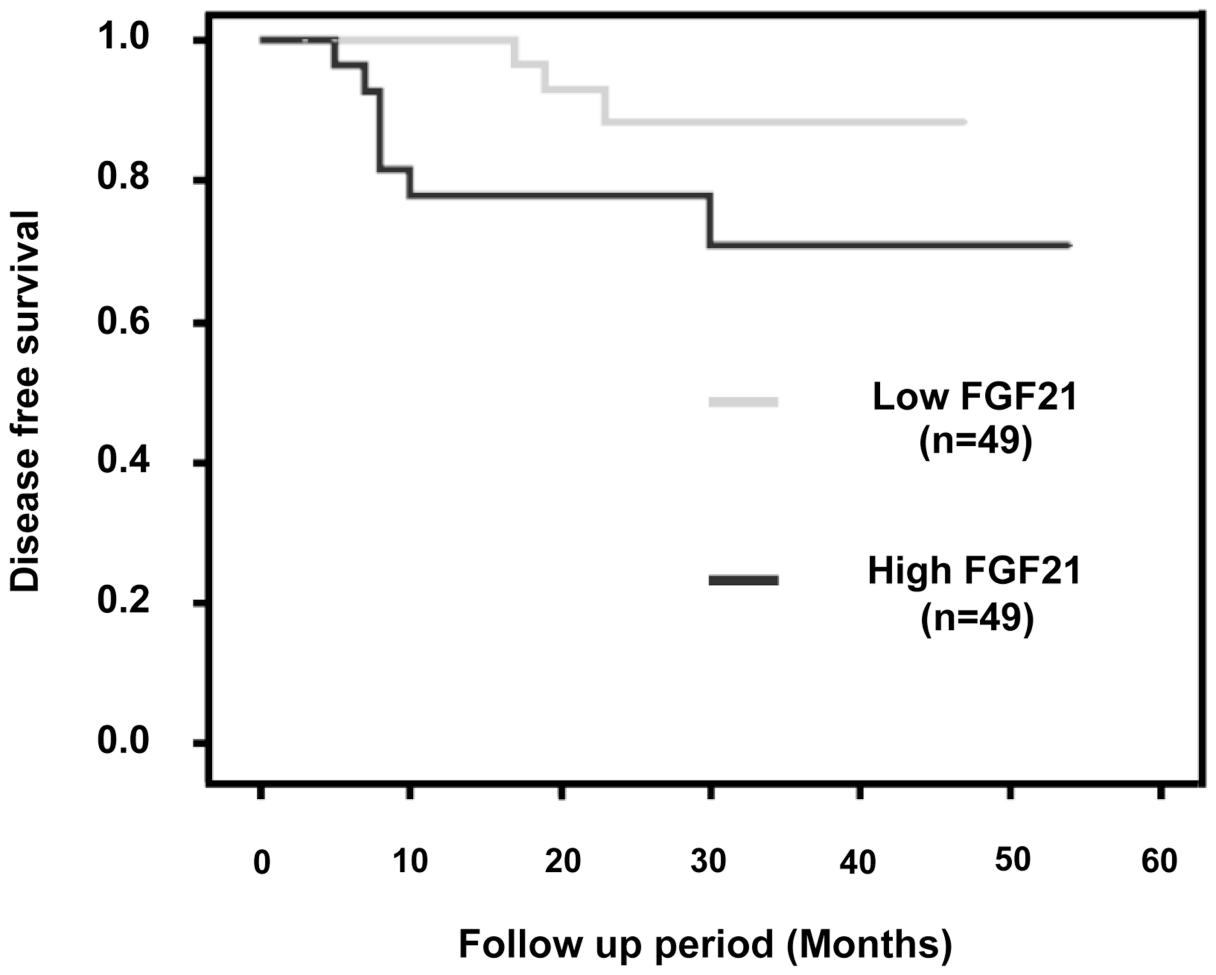
Kaplan-Meier survival curve for the association between FGF21 serum levels and disease-free survival (DFS) for ccRCC patients. Patients were categorized as low-FGF21 and high-FGF21 expression groups according to the optimal cut-off value. Survival analysis was performed using Kaplan-Meier analysis, with the differences between curves analyzed via a long-rank test (Log-Rank test: 3.28, p=0.07).

Then, we analyzed whether FGF21 levels had an impact on disease-free survival (DFS). The Kaplan-Meier plots of DFS showed that high levels of serum FGF21 were associated with worse prognosis with a borderline significance (Log-Rank test: 3.28, p=0.07) (Figure 6). This borderline significance enabled us to perform a multivariate test. Surprisingly, when we evaluated the independence of the clinico-pathological parameters on DFS (Cox Regression test), we found that FGF21 expression is an independent prognostic factor when adding the variables Fuhrman grade and stage (data not shown).

**Figure 7:**
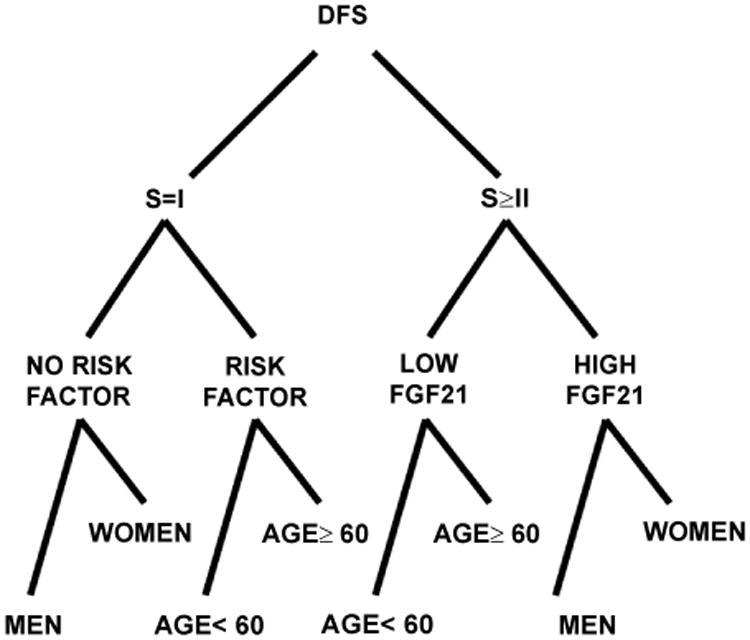
Decision tree analysis to determine the effect of relevant clinico-pathological parameters on disease free survival (DFS) of ccRCC. Variables are represented as nodes that sequentially split according to those with the highest effect on variation in data. S: Stage. For a better visualization only node I, II and III are shown.

Finally, we evaluated the effect of specific variables on DFS, including the known predictors of ccRCC prognosis (stage, age, sex, risk factors, Fuhrman grade) and the serum FGF21 levels. We performed an additional multivariate analysis by creating a decision tree-structured model. In this model, the initial split was made on the stage. However, FGF21 differentiated DFS on node II, being Stage II and III patients with high levels of FGF21 those with worse clinical outcome, in terms of DFS (Table 3) (Figure 7).

### Serum FGF21 in another CCR population

We also measured circulating FGF21 levels in patients with other types of renal cancer. Interestingly, serum FGF21 levels were significantly increased in patients with chromophobe renal cancer (n=14; Md (range): 236.59 pg/ml (125.71-1195.40)); (MW test: p<0.0001) respect to the HC. Serum FGF21 levels were similar in patients suffering from clear cell or chromophobe pathology (MW test, NS).

## Discussion

In this study, we measured the serum FGF21 levels in ccRCC patients and evaluated its potential value as a diagnostic and prognostic biomarker in this disease. Our results showed an association between the increased serum FGF21 levels and the shorter DFS in a cohort of 98 ccRCC patients, after adjustment for other predictors of outcome. Inspite of the fact that ccRCC is a rare type of cancer; our limited number of cases and controls, allowed us to demonstrate that FGF21 levels were significantly increased in ccRCC patients compared with HC. This increase was observed since the onset of the disease but no difference was observed among stages, which reinforces its potential effectiveness as a renal cancer diagnostic biomarker.

At first, we showed that the median serum FGF21 level in our study was similar to previously reported values [25,26].

It was reported that serum FGF21 correlated positively with triglycerides but negatively with HDL and cholesterol, after adjusting for age and BMI, in a population that included obese and lean individuals [27]. Our study showed that HC's serum FGF21 levels were neither associated to age or gender nor correlated to LDL, HDL, cholesterol or glycemia levels. However, serum FGF21 levels significantly correlated with the triglycerides levels, in an independent way. In this study, we demonstrated that leptin and FGF21 were not associated. In addition, FGF21 was no associated with age, gender, glycemia, LDL, HLD or total cholesterol. Despite that the triglycerides levels were similar in ccRCC and HC populations, our cohort of cancer patients showed no correlation between FGF21 and triglycerides. Moreover, ccRCC patients showed no correlation with BMI or body weight.

Serum FGF21 showed a high sensibility to diagnose ccRCC (∼80%) although with a medium specificity (∼65%). Serum FGF21 levels were also increased in patients with chromophobe RCC, another histological type of RCC, demonstrating that this increase is not restricted to ccRCC patients.

Serum FGF21 levels could also be induced by other clinical conditions such as fasting, obesity, liver injury, cirrhosis, renal dysfunction, metabolic syndrome, type 2 diabetes and coronary disease [9,11,19,20,27-29]. In particular, increased FGF21 serum levels produced by the liver under stressful conditions may serve as an inter-organ feedback communication to minimize the stress-induced damage. In our study, we did not obtain any association between the values of creatinine clearance and FGF21 levels, suggesting that the elevated serum FGF21 levels were not due to a renal dysfunction.

There is no information available on the role of FGF21 in tumor initiation and progression; further studies are needed to elucidate the mechanisms behind the increased serum FGF21 levels in renal cancer patients. The elevated FGF21 levels observed in these patients could be due to a high FGF21 synthesis by the tumor cells or the stress caused microenvironment metabolic disorders. Our results showed increased serum FGF21 levels since the early stages of the disease but no differences were observed among stages. These findings suggest that the initiation of the tumor itself could be considered as a stressful condition that induces an increased FGF21 secretion by hepatocytes or/and adipocytes. Supporting this hypothesis, our analysis of FGF21 levels after resection of the primary tumor, showed that FGF21 remained highly expressed in 50% of ccRCC patients during early post-surgical follow-up (data not shown). However, we cannot rule out that the subclinical disease may explain the lack of association during early follow-up.

On the other hand, it is known that circulating FGF21 levels enhance insulin action [30] and lead to an increased glucose uptake by the peripheral tissues. Other hypothesis, which deserves further investigation, is that the growing tumor induces FGF21-mediated insulin secretion which leads to an increased uptake of glucose by the tumor to sustain its energy requirements and growth.

Considering the information discussed, it is highly possible that FGF21 could be a universal cancer biomarker rather than a specific diagnosis biomarker for ccRCC, and it should be used in combination with other existing biomarkers.

Despite all the current prognostic and predictive biomarkers in RCC, there are currently several obstacles for their application into the clinic. There is a clear need to further incorporate molecular markers in clinical decisions.

Up-to-date; there is no previous published data on the role of FGF21 as prognostic biomarker. Our results indicated that FGF21 is a promising prognosis biomarker. In addition, there are no so many clinical studies in non-clear cell histology's biomarkers due to their lower prevalence. Therefore, one important additional direction of this study was to evaluate the circulating FGF21 levels in non-clear cell histology tumors.

We showed that ccRCC patients with increased FGF21 levels at diagnosis had a shorter relapse free survival with a borderline significance. Moreover, the Cox multivariate regression model indicated that FGF21 is an independent DFS biomarker not related with stage or Fuhrman nuclear grade, the two clinical prognostic biomarkers employed currently in ccRCC. In addition, by creating a decision tree, FGF21 differentiated DFS for patients with higher stages on node II (after the variable “Stage”, in node I).

In summary, our results indicate that high serum FGF21 levels are associated with worse prognosis in terms of relapse in the follow-up period. Furthermore, we believe that the availability of molecular biomarkers such as FGF21 will help to facilitate patient management and further improve the clinical outcome.

## Figures and Tables

**Table 1:** Age and gender characterization of HC and patient populations.

		n	Age	
			Median	Range
HC	Men	25	42	23-63
	Women	26	44.5	20-63
ccRCC	Men	69	61	42-85
	Women	29	57.5	43-75
RCC chromophobe	Men	8	55	23-71
	Women	6	57.5	31-75

**Table 2:** Association between the serum FGF21 levels and clinico-pathological parameters of ccRCC patients.

Parameter		FGF21	Χ^2^ Test, p value
		High/Total (%)	
Sex (n=98)	M	33/70 (47.1)	p=0.27
	W	16/28 (57.1)	
Age (n=98)	<60	24/48 (50.0)	P=1.00
	≥60	25/50 (50.0)	
Obesity (n=95)	No	42/82 (51.2)	p=0.39
	Yes	5/13 (38.5)	
Risk Factors (n=92)	No	10/21 (47.6)	p=0.89
	Ye s	35/71 (49.3)	
TG (n=30)	High	2/3 (66.7)	p=0.9
	Low	17/27 (63.0)	
Stage (n=98)	I	19/37 (51.4)	p=0.68
	II	5/14 (35.7)	
	III	10/20 (50.0)	
	IV	15/27 (55.6)	
Fuhrman Grade (n=94)	1	1/3 (3.3)	p=0.77
	2	11/25 (44.0)	
	3	20/43 (46.5)	
	4	13/23 (56.5)	
Tumor Size (n=95)	1	23/45 (51.5)	p=0.57
	2	7/19 (36.8)	
	3	13/25 (52.0)	
	4	4/6 (66.7)	
Metastasis (n=98)	No	35/73 (47.9)	p=0.49

**Table 3:** Classification tree analysis for the serum FGF21 levels and some prognosis predictors.

Variable	DFS							
First node	Stage=I				Stage ≥ II			
Second node	Risk Factor=No		Risk Factor=Yes		FGF21=“low”		FGF21=“high”	
Third node	Sex=M	Sex=F	Age<60	Age ≥ 60	Age <60	Age ≥ 60	Sex= M	Sex=F

## Abbreviations

FGF21: Fibroblast Growth Factor 21
ccRCC: clear-cell Renal Cell Carcinoma
HC: Healthy Controls
DFS: Disease-Free Survival
OS: Overall Survival
